# Antibody Responses 8 Months after Asymptomatic or Mild SARS-CoV-2 Infection

**DOI:** 10.3201/eid2703.204543

**Published:** 2021-03

**Authors:** Pyoeng Gyun Choe, Kye-Hyung Kim, Chang Kyung Kang, Hyeon Jeong Suh, EunKyo Kang, Sun Young Lee, Nam Joong Kim, Jongyoun Yi, Wan Beom Park, Myoung-don Oh

**Affiliations:** Seoul National University College of Medicine, Seoul, South Korea (P.G. Choe, C.K. Kang, H.J. Suh, E. Kang, S.Y. Lee, N.J. Kim, W.B. Park, M.-d. Oh);; Pusan National University School of Medicine, Busan, South Korea (K.-H. Kim, J. Yi)

**Keywords:** 2019 novel coronavirus disease, coronavirus disease, COVID-19, severe acute respiratory syndrome coronavirus 2, SARS-CoV-2, viruses, respiratory infections, zoonoses, antibody, ELISA, ECLIA

## Abstract

Waning humoral immunity in coronavirus disease patients has raised concern over usefulness of serologic testing. We investigated antibody responses of 58 persons 8 months after asymptomatic or mildly symptomatic infection with severe acute respiratory syndrome coronavirus 2. For 3 of 4 immunoassays used, seropositivity rates were high (69.0%–91.4%).

Infection with severe acute respiratory syndrome coronavirus 2 (SARS-CoV-2) leads to an antibody response, even in those who are completely asymptomatic. However, the initial immune response is not as strong as in patients with more severe disease, and concerns about waning immunity have been raised (*1*,*2*). We evaluated the antibody responses of 58 persons in South Korea 8 months after asymptomatic or mildly symptomatic SARS-CoV-2 infection.

## The Study

The eligible participants for this cross-sectional survey were persons with reverse transcription PCR–confirmed coronavirus disease (COVID-19) who had been isolated in a community treatment center (CTC) operated by Seoul National University Hospital during March 5–April 9, 2020. Isolation was in response to the COVID-19 outbreak in Daegu, South Korea (population 2.4 million), the first large outbreak outside of China, which resulted in 6,620 confirmed cases during February 18–March 24, 2020 (*3*). CTC admission criteria were as follows: alert, age <65 years, no underlying disease or well-controlled underlying disease, body temperature <38.0°C with or without antipyretics, and no dyspnea. During participants’ CTC stay, physicians and nurses comprehensively evaluated them twice daily via video consultation. Asymptomatic persons were defined as those with body temperature <37.5°C and no signs or symptoms (e.g., no subjective fever, myalgia, rhinorrhea, sore throat, cough, sputum, or chest discomfort) during the entire CTC stay; others were classified as mildly symptomatic patients (*4*). From all participants who provided informed consent, we collected serum samples at 8 months after infection.

We measured SARS-CoV-2–specific antibodies by using 4 commercial immunoassays: an antinucleocapsid (anti-N) panimmunoglobulin (pan-Ig) electrochemiluminescence immunoassay (ECLIA) (Elecsys Anti-SARS-CoV-2; Roche Diagnostics, https://diagnostics.roche.com), an anti-N IgG ELISA (EDI Novel Coronavirus COVID-19 ELISA Kit; Epitope Diagnostics, https://www.epitopediagnostics.com), an antispike (anti-S) IgG ELISA (SCoV-2 Detect IgG ELISA; InBios International, https://www.inbios.com), and an anti-S1 spike subunit IgG ELISA (Anti-SARS-CoV-2 ELISA (IgG); Euroimmun, https://www.euroimmune.com). Except for the anti-N IgG ELISA, these immunoassays were granted Emergency Use Authorization by the US Food and Drug Administration. Measurement and interpretation of results were made according to each manufacturer’s instructions. For the anti-N and anti-S1 IgG ELISAs, borderline results were regarded as negative. To evaluate neutralizing activity targeting the spike receptor–binding domain, we used a surrogate virus neutralization test (sVNT) (SARS-CoV-2 Surrogate Virus Neutralization Test; GenScript, https://www.genscript.com) (*5*). The Institutional Review Boards of Seoul National University Hospital and the Pusan National University Hospital approved the study (IRB nos. H-2009-168-1160 and H-2010-013-096).

We analyzed data from 7 participants with asymptomatic SARS-CoV-2 infection and 51 patients with mildly symptomatic COVID-19 (Table 1). Eight months after their infections, we detected anti-N pan-Ig in 53 (91.4%), anti-N IgG in 15 (25.9%), anti-S IgG in 50 (86.2%), and anti-S1 IgG in 40 (69.0%) (p<0.01) (Table 2). The sVNT found positive neutralizing activity for 31 (53.4%). For female participants, positivity was significantly higher for anti-N IgG (40.0% female vs. 4.3% male; p<0.01), anti-S IgG (94.3% vs. 73.9%; p<0.05), anti-S1 IgG (82.9% vs. 47.8%; p<0.01), and sVNT (68.6% vs. 30.4%; p<0.01). Positivity by PCR for <14 days was associated with a lower rate of positivity for anti-N pan-Ig (50.0% for <14 d vs. 96.0% for >14 d; p<0.01) (Table 2). Logistic regression analysis, for which anti-N IgG ELISA results were excluded because of exceptionally low positivity, indicated that negative results from >2 commercial immunoassays were significantly associated with positivity by PCR for <14 days after adjustment for sex (adjusted odds ratio 11.49; 95% CI 1.45–90.79; p = 0.02) (Appendix).

**Table 1:** Clinical characteristics of 58 persons with asymptomatic or mildly symptomatic severe acute respiratory syndrome coronavirus 2 infection, South Korea*

Characteristic	Asymptomatic	Mildly symptomatic
Total no. persons	7	51
Sex, no. (%)		
M	5 (71.4)	18 (35.3)
F	2 (28.6)	33 (64.7)
Age, y, median (IQR)	25 (21–26)	26 (22–40)
Underlying disease, no. (%)†	0	3 (5.9)
Smoking status		
Smoker	0	0
Ex-smoker	1 (14.3)	2 (3.9)
Nonsmoker	6 (85.7)	49 (96.1)
Signs/symptoms, no. (%)		
Febrile/chilling sense	NA	8 (15.7)
Myalgia	NA	5 (9.8)
Headache	NA	13 (25.5)
Cough	NA	20 (39.2)
Sputum	NA	33 (64.7)
Rhinorrhea	NA	25 (49.0)
Sore throat	NA	3 (5.9)
Chest discomfort/dyspnea	NA	4 (7.8)
Duration of PCR positivity, d, median (IQR)	29 (25–34)	24 (19–34)
Days from symptom onset to blood sampling, median (IQR)‡	231 (231–233)	234 (231–234)

*IQR, interquartile range; NA, not applicable. †One each: hypertension, diabetes mellitus, asthma. ‡For asymptomatic patients, time from the first PCR-positive result to blood sampling.

**Table 2:** Positivity of antibodies to severe acute respiratory syndrome coronavirus 2 in 58 asymptomatic or mildly symptomatic patients at 8 mo after infection, South Korea*

Characteristic	Anti-N pan-Ig ECLIA, no. (%)†	Anti-N IgG ELISA, no. (%)‡	Anti-S IgG ELISA, no. (%)§	Anti-S1 IgG ELISA, no. (%)¶	sVNT, no. (%)#
Total	53/58 (91.4)	15/58 (25.9)	50/58 (86.2)	40/58 (69.0)	31/58 (53.4)
Sex					
M	21/23 (91.3)	1/23 (4.3)	17/23 (73.9)	11/23 (47.8)	7/23 (30.4)
F	32/35 (91.4)	14/35 (40.0)**	33/35 (94.3)††	29/35 (82.9)**	24/35 (68.6)**
Age, y					
<30	35/38 (92.1)	5/38 (13.2)	33/38 (86.8)	25/38 (65.8)	19/38 (50.0)
>30	18/20 (90.0)	10/20 (50.0)**	17/20 (85.0)	15/20 (75.0)	12/20 (60.0)
Duration of PCR positivity					
<14 d	3/6 (50.0)	3/6 (50.0)	4/6 (66.7)	3/6 (50.0)	2/6 (33.3)
>14 d	48/50 (96.0)**	10/50 (20.0)	44/50 (88.0)	36/50 (72.0)	27/50 (54.0)
Disease severity					
Asymptomatic	7/7 (100)	0/7 (0)	5/7 (71.4)	4/7 (57.1)	4/7 (57.1)
Mildly symptomatic	46/51 (90.2)	15/51 (29.4)	45/51 (88.2)	36/51 (70.6)	27/51 (52.9)

*Anti-N, antinucleocapsid; anti-S, antispike; anti-S1, antispike subunit; ECLIA, electrochemiluminescence immunoassay; pan-Ig, panimmunoglobulin; sVNT, surrogate virus neutralization test. †Roche Diagnostics (https://diagnostics.roche.com).  ‡Epitope Diagnostics (https://www.epitopediagnostics.com). §InBios International (https://www.inbios.com). ¶Euroimmun (https://www.euroimmune.com). #GenScript (https://www.genscript.com). **p<0.01. ††p<0.05.

## Conclusions

Knowledge of the longevity of humoral immunity to SARS-CoV-2 is essential for predicting herd immunity and interpreting seroepidemiologic data. Recent studies showed that the antibody titers of patients with mild SARS-CoV-2 infection declined more quickly than those reported for SARS-CoV patients (*6*), and waning immunity was confirmed 5 months after infection (*7*). Concern about the usefulness of population-based seroprevalence studies has been raised because rapidly waning immunity may lead to a substantial number of false-negative immunoassay results (*2*). However, in this study, we confirmed that rates of antibody positivity according to 3 commercial kits was still high at 8 months after infection, even in asymptomatic or mildly symptomatic participants (69.0%–91.4%). Rates differed according to immunoassay methods or manufacturers, thereby explaining differences in rates between the studies (*2*,*8*). A previous study argued that among asymptomatic persons who had been antibody positive early in the infection, 40% became antibody negative in 2–3 months, even when tested by chemiluminescence immunoassay (CLIA) (*2*); however, their results are in stark contrast to ours, which may have resulted from variations in the characteristics of CLIA products from different manufacturers. In a systematic review and meta-analysis, pooled sensitivity was 97.8% with CLIA in contrast to 84.3% with ELISA (*9*). In a head-to-head benchmark comparison study, anti-N pan-Ig ECLIA showed 97.2% sensitivity and 99.8% specificity (*10*). In the previous studies, CLIA showed high sensitivity and specificity for recent or past SARS-CoV-2 infection. Therefore, our results show that a serosurvey is useful even 8 months after an outbreak if an appropriate binding immunoassay format like an anti-N pan-Ig ECLIA is used. A serosurvey that uses a binding immunoassay can determine the infected proportion of the population and also the proportion of infections detected by PCR, thus enabling inference of the infection-fatality ratio rather than just the case-fatality ratio; however, it cannot accurately assess population immunity because it is not a functional immunoassay for detecting neutralizing activity.

Neutralizing activity, a functional aspect of antibodies, is essential for protection from reinfection and screening potential convalescent plasma therapy donors (*8*). In our study, neutralizing activity was detected in 53.4% of asymptomatic or mildly symptomatic participants after 8 months of infection, which was considerably lower than the rate of positivity detected by binding immunoassays such as ECLIA or ELISAs. This finding is not surprising because neutralizing activity is affected by various factors, including the antigen specificity and the amount of existing antibody. However, confirming sVNT results by conventional VNT might be needed, although the reported specificity (100%) and sensitivity (98%–98.9%) of sVNT showed good correlation with conventional VNT (*5*). A recently published study of convalescent plasma therapy found detectable neutralizing antibodies in 63.6% persons a median of 41 days after PCR-confirmed diagnosis of mild COVID-19 (*11*).

According to our study, prolonged duration of virus shedding is associated with long-term antibody positivity in patients with mild COVID-19, which aligns with previous findings of higher IgG levels during weeks 4–8 in those in the prolonged virus shedding group (*12*). Factors associated with prolonged virus shedding include male sex, old age, severe illness at admission, and invasive mechanical ventilation (*13*). Our findings suggest that the duration of virus shedding reflects the amount of humoral immune stimulation, even in asymptomatic or mildly symptomatic persons with COVID-19.

One limitation of our study was the relatively small sample size and the predominantly young population, which lessen generalization of the results. Also, because of the cross-sectional design, we could not obtain baseline or longitudinal serum samples. For the 7 asymptomatic participants in our study, we evaluated antibody responses at 2 and 5 months after infection; 5/7 (71%) had positive ELISA results at 2 months after infection, 4/7 (57.1%) had positive ELISA results at 5 months after infection, and all had neutralizing antibodies at 2 and 5 months after infection (*1**,**7*). Last, we could not assess the individual possibilities of reexposure or reinfection. However, it is unlikely that humoral immunity was boosted because in Daegu, where the study participants reside, during April–October 2020, the daily incidence rate for COVID-19 was <0.5 cases/100,000 population (*14*). In conclusion, despite concerns of waning immunity, appropriate immunoassays can detect antibodies against SARS-CoV-2 at 8 months after infection in most asymptomatic or mildly symptomatic persons.

Appendix Logistic regression analysis of negativity detected by >2 commercial kits for 58 patients 8 months after asymptomatic or mildly symptomatic infection with severe acute respiratory syndrome coronavirus 2, South Korea.
