# Supplementary material for: Antibody Responses 8 Months after Asymptomatic or Mild SARS-CoV-2 Infection
Source: Emerg Infect Dis. 2021 Mar;27(3):928–31. doi: 10.3201/eid2703.204543 (PMC7920668; doi:10.3201/eid2703.204543)
Supplement: Appendix — Logistic regression analysis of negativity detected by >2 commercial kits for 58 patients 8 months after asymptomatic or mildly symptomatic infection with severe acute respiratory syndrome coronavirus 2, South Korea. [file 20-4543-Techapp-s1.pdf]

Article DOI: https://doi.org/10.3201/eid2703.204543

Antibody Responses 8 Months after Asymptomatic or Mild SARS-CoV-2 Infection

Appendix

Appendix Table. Logistic regression analysis for negativity in two or more commercial kits at 8 mo after infection in 58 asymptomatic or mildly symptomatic patients with severe acute respiratory syndrome coronavirus 2 infection*

	Univariate analysis			Multivariate analysis		
Factor	OR	95% CI	p-value	aOR	95% CI	p-value
Age						
>30 y	1.00					
≤30 y	1.06	0.23–4.78	0.937			
Sex						
F	1.00					
M	3.76	0.83–16.96	0.084	5.28	0.93–29.75	0.059
Duration of PCR positivity						
>14 d	1.00					
≤14 d	7.66	1.25–46.95	0.028	11.49	1.45–90.79	0.021
Disease severity						
Mildly symptomatic	1.00					
Asymptomatic	2.51	0.45–15.57	0.322			

*OR, odd ratio; aOR, adjusted odd ratio; CI, confidence interval.
